# Supplementary material for: Multi-Omics Integrative Analyses Identified Two Endotypes of Hip Osteoarthritis
Source: Metabolites. 2024 Sep 1;14(9):480. doi: 10.3390/metabo14090480 (PMC11434176; doi:10.3390/metabo14090480)
Supplement: Supplementary file 1 [file metabolites-14-00480-s001.zip › Supplementary figure.pdf]

Figure S1. Results of principal component analysis (PCA) for batch effects checking

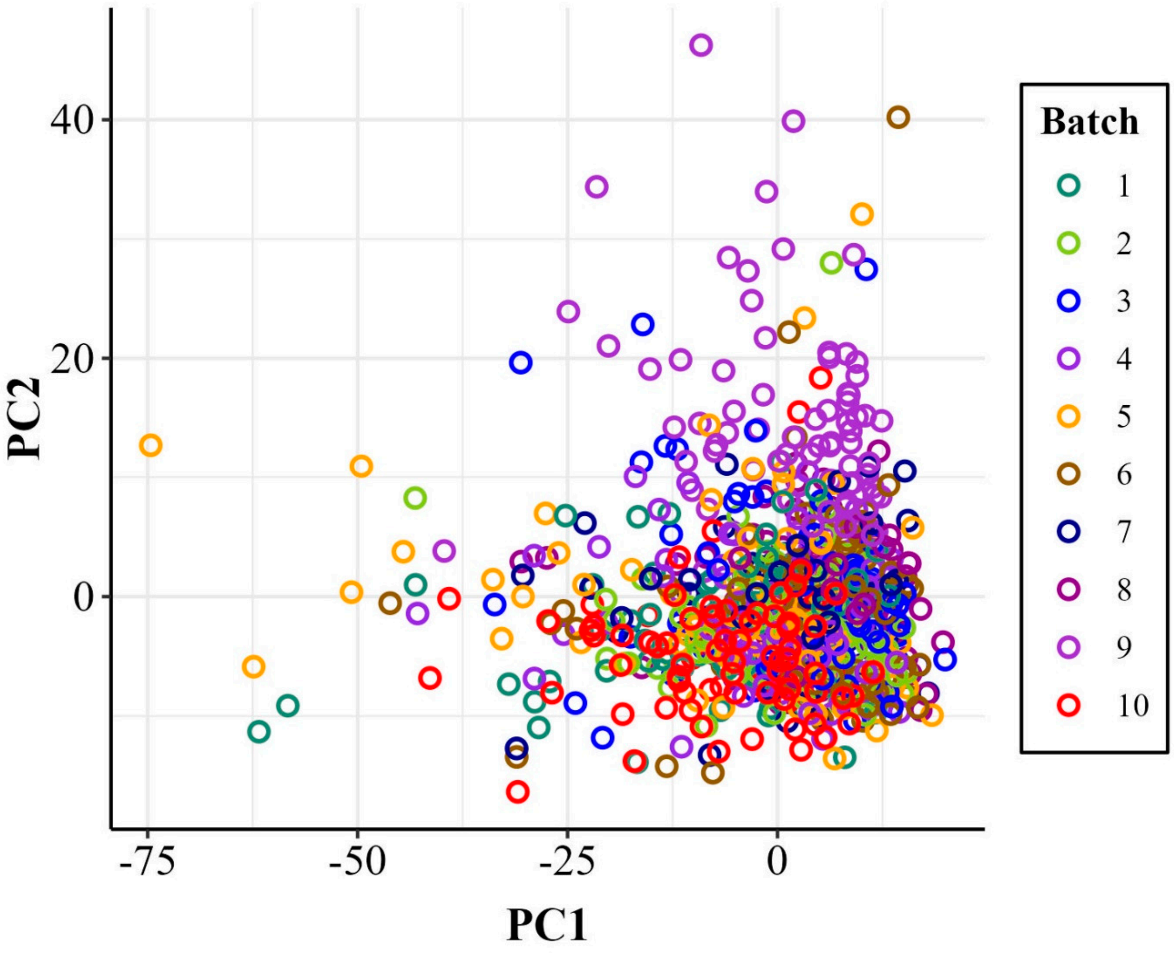

Different test plates were marked with different colors, and each dot represents a sample. No batch effect was detected.

Figure S2. Robustness validation result

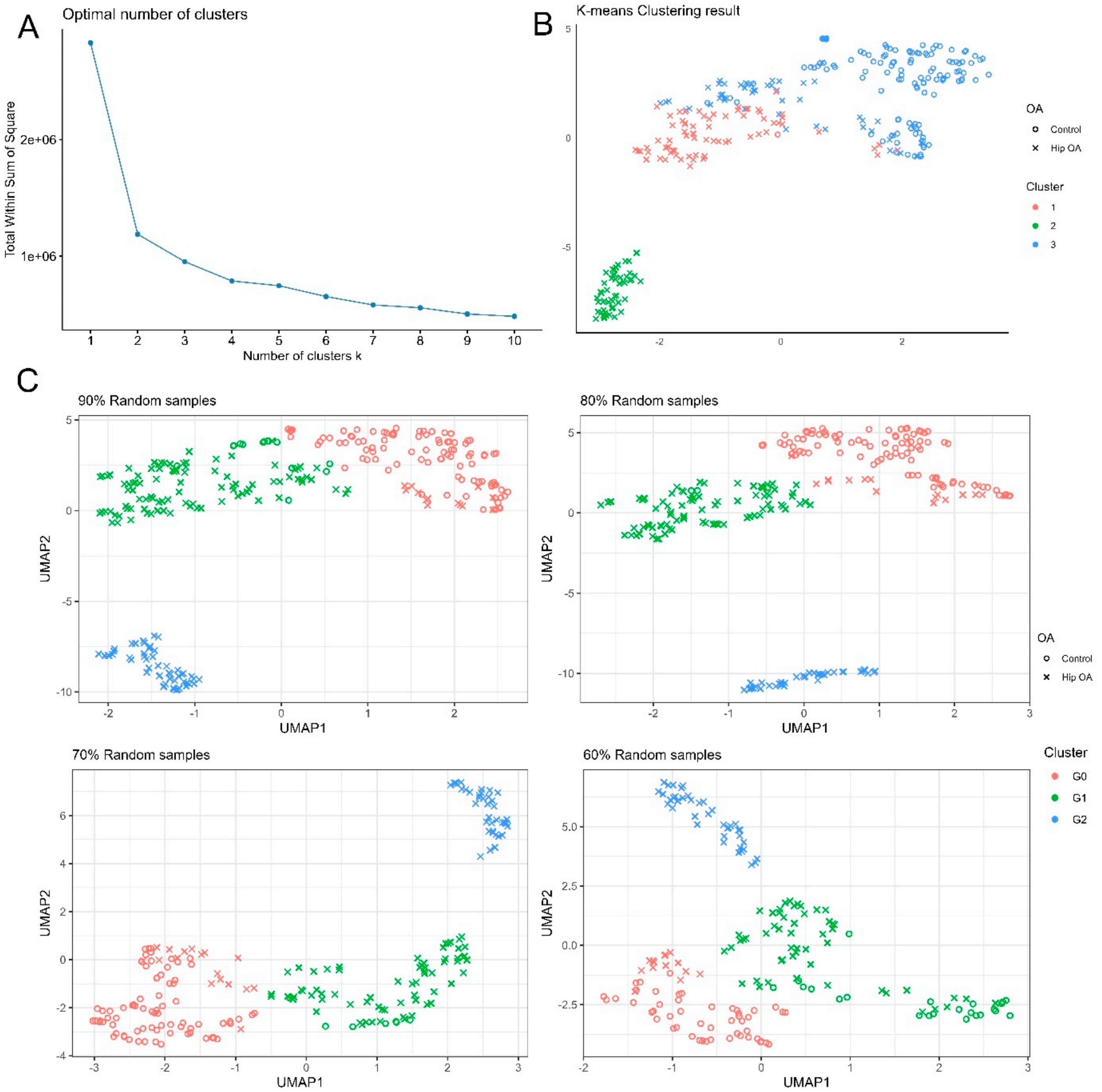

(A) The elbow plot estimates that 300 participants can be divided into three main groups based on metabolomic data. (B) The clustering results obtained by K-means was mapped to the UMAP clustering results. (C) The UMAP results of different proportions of the randomly drawn samples from the entire cohort.

Figure S3. Venn diagram showing the overlap between the three pathway lists

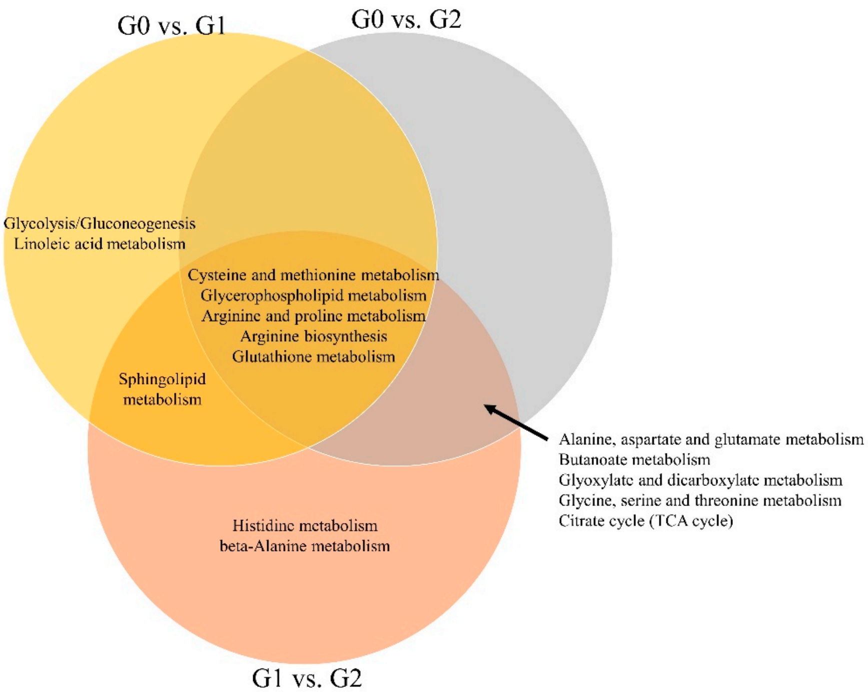

Figure S4. The Manhattan plot of Genome-wide association studies (GWAS) results of seven metabolites

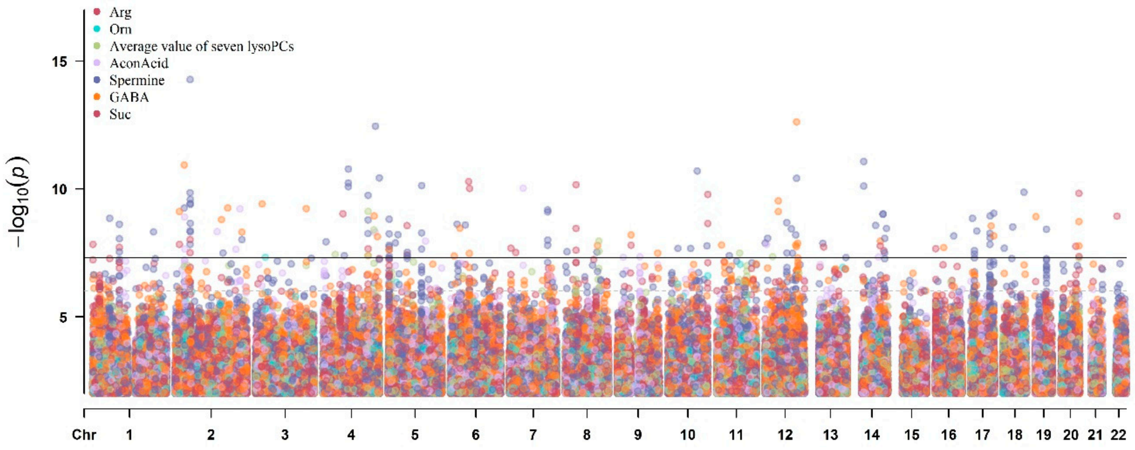

GWAS significant level is P < 5×10^{-8} as indicated by the horizontal line.

Figure S5. Quantile-quantile (QQ) plots for each identified metabolites in Genome-wide association studies (GWAS) analyses

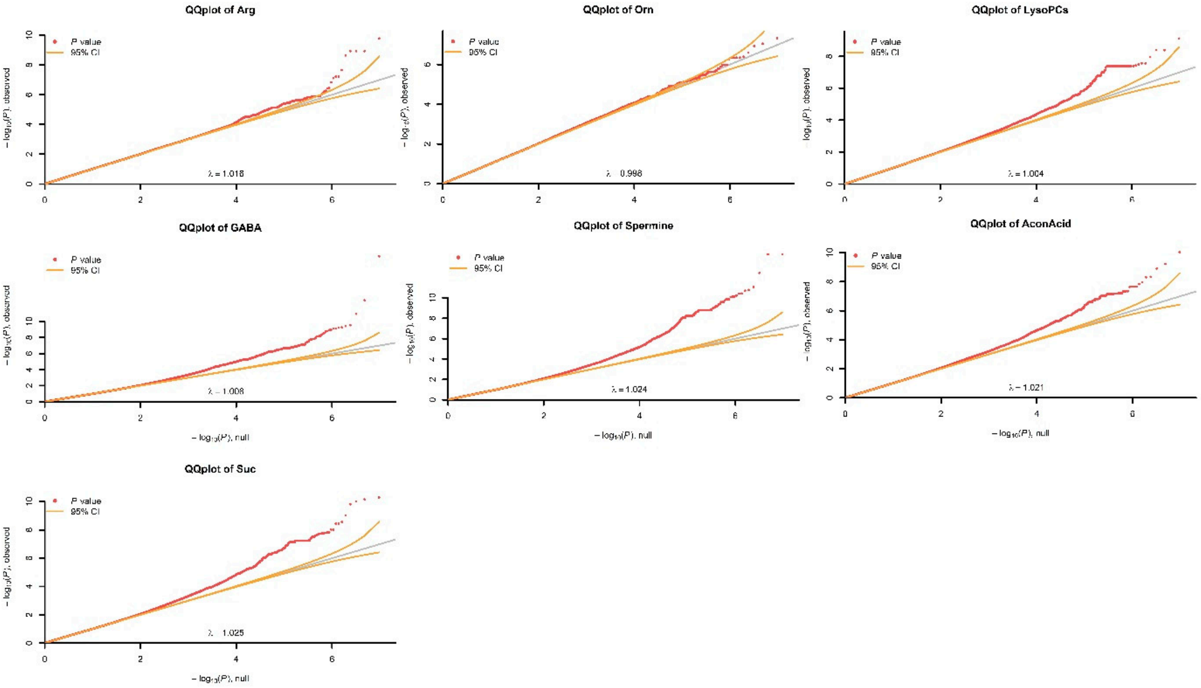

Figure S6. GO and KEGG enrichment analyses results

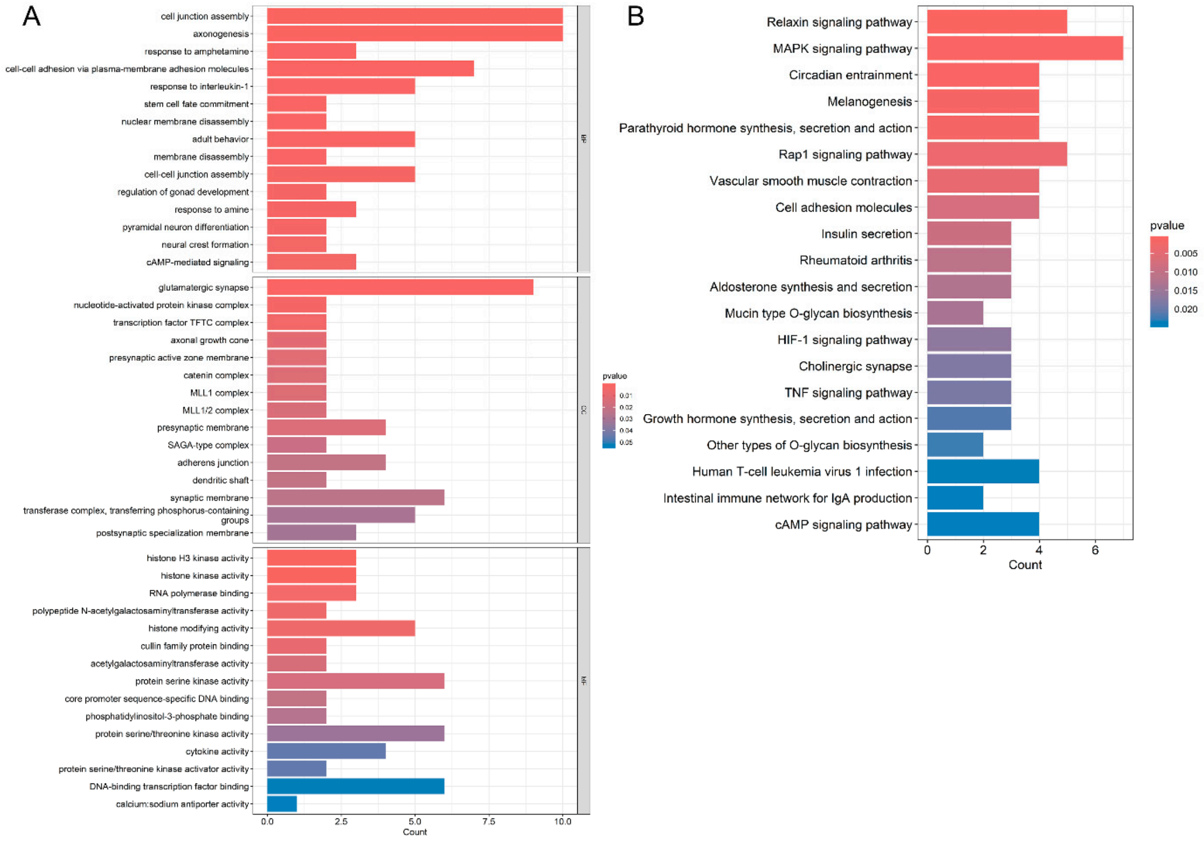

(A) Gene Ontology (GO) enrichment analysis conducted on 130 identified genes.

(B) Kyoto Encyclopedia of Genes and Genomes (KEGG) enrichment analysis conducted on 130 identified genes.
